# Climate change impact on seaweed meadow distribution in the North Atlantic rocky intertidal

**DOI:** 10.1002/ece3.541

**Published:** 2013-04-12

**Authors:** Alexander Jueterbock, Lennert Tyberghein, Heroen Verbruggen, James A Coyer, Jeanine L Olsen, Galice Hoarau

**Affiliations:** 1 Faculty of Biosciences and Aquaculture, University of Nordland8049, Bodø, Norway; 2 Flanders Marine Institute VLIZWandelaarkaai 7, 8400, Oostende, Belgium; 3 Phycology Research Group, Biology Department, Ghent UniversityKrijgslaan 281, 9000, Ghent, Belgium; 4 School of Botany, University of MelbourneVictoria, 3010, Australia; 5 Shoals Marine Laboratory, Cornell UniversityPortsmouth, New Hampshire, 03801; 6 Marine Benthic Ecology and Evolution Group, Centre for Ecological and Evolutionary Studies, University of GroningenNijenborgh 7, 9747 AG, Groningen, The Netherlands

**Keywords:** *Ascophyllum*, ecological niche models, *Fucus*, geographic distribution, global warming, intertidal, macroalgae, species distribution models

## Abstract

The North-Atlantic has warmed faster than all other ocean basins and climate change scenarios predict sea surface temperature isotherms to shift up to 600 km northwards by the end of the 21st century. The pole-ward shift has already begun for many temperate seaweed species that are important intertidal foundation species. We asked the question: Where will climate change have the greatest impact on three foundational, macroalgal species that occur along North-Atlantic shores: *Fucus serratus*, *Fucus vesiculosus,* and *Ascophyllum nodosum*? To predict distributional changes of these key species under three IPCC (Intergovernmental Panel on Climate Change) climate change scenarios (A2, A1B, and B1) over the coming two centuries, we generated Ecological Niche Models with the program MAXENT. Model predictions suggest that these three species will shift northwards as an assemblage or “unit” and that phytogeographic changes will be most pronounced in the southern Arctic and the southern temperate provinces. Our models predict that Arctic shores in Canada, Greenland, and Spitsbergen will become suitable for all three species by 2100. Shores south of 45° North will become unsuitable for at least two of the three focal species on both the Northwest- and Northeast-Atlantic coasts by 2200. If these foundational species are unable to adapt to the rising temperatures, they will lose their centers of genetic diversity and their loss will trigger an unpredictable shift in the North-Atlantic intertidal ecosystem.

## Introduction

### Species responses to climate change

Studies on the global response of a wide variety of marine and terrestrial species to climate change conclude that the planet is facing drastic ecosystem shifts and numerous extinctions (Hughes 2000; Davis and Shaw 2001; Parmesan and Yohe 2003; Root et al. 2003; Parmesan 2006; Rosenzweig et al. 2008). Species that fail to acclimatize physiologically or evolve genetically to increasing temperatures will either move northwards into cooler habitats (Walther et al. 2002; Parmesan and Yohe 2003; Parmesan 2005, 2006; Hickling et al. 2006; Thomas 2010) or become extinct (Thomas et al. 2004).

Responses to climate change are particularly rapid and strong in marine ecosystems (Southward et al. 1995; Hoegh-Guldberg and Bruno 2010; Sorte et al. 2010), especially in the marine intertidal where species often live at their upper temperature tolerance limits (Somero 2010). Global warming-related range shifts of marine species (on average 19 km/year Sorte et al. 2010) exceed those of terrestrial species (0.6 km/year Parmesan and Yohe 2003) by an order of magnitude. Furthermore, climate-change induced range shifts are more predictable for marine than for terrestrial species, since the distributional limits of marine species are usually directly correlated with their thermal tolerance limits (Sunday et al. 2012). Range shifts of key or foundation species are of central importance, since by definition these species play a crucial role in, and can trigger changes throughout, the entire web of interactions within an ecological community (Kordas et al. 2011).

### Climate change threatens seaweed meadows on temperate rocky shores

Canopy-forming macroalgae are foundation species (sensu Dayton 1972), playing a pivotal role in rocky intertidal communities of temperate shores (e.g., Hicks 1964; Edgar and Moore 1986; Fredriksen et al. 2005). They increase the habitable surface by at least a factor of four (Boaden 1996) and provide food and habitat that support a complex food web (Carss and Elston 2003; Gollety et al. 2010). Algal canopies also dampen extreme temperature and salinity oscillations over a tidal/seasonal cycle; facilitate invertebrate recruitment and growth; and provide protection from wave action, desiccation, and visual predators (reviewed in Chapman 1995; Wahl et al. 2011; Dijkstra et al. 2012). Furthermore, macroalgae beds form a substantial sink for CO_2_ emissions (Gao and McKinley 1994; Muraoka 2004; Chung et al. 2011), sequestering about 1 gigaton of carbon (GtC) year^−1^ (together with sea grass beds) (Gao and McKinley 1994; Chung et al. 2011), which equals about a quarter of the current yearly atmospheric carbon increase (4.1 ± 0.1 GtC; Denman et al. 2007).

The seaweed community characterizing the phytogeographic temperate region of the North-Atlantic (ca. 40°N–50°N on the Northwest-Atlantic and ca. 20°N to 70°N on the NE coast) (Van den Hoek 1975) differs markedly from the adjacent polar (north of the 15°C summer isotherm) and tropical regions (south of the 20°C winter isotherm) (Van den Hoek 1975; Lüning et al. 1990). Toward the southern warm-temperate region, barnacles and intertidal grazers, as well as green and red algae, replace canopy-forming seaweed meadows (Lüning et al. 1990; Southward et al. 1995; Lima et al. 2007). Toward the Arctic region, seaweed diversity decreases and the macroalgal flora is primarily confined to the subtidal (Van den Hoek 1975; Wiencke and Amsler 2012).

Temperature profoundly influences the survival, recruitment, growth, and reproduction of seaweeds (Breeman 1988). Thus, seaweed distributions are correlated with sea surface temperature (SST) isotherms (Lüning et al. 1990) and likely will respond directly to climate change with range shifts: extinction at the southern and colonization at the northern boundaries. With a temperature increase from 0.4°C to 1.6°C from the mid-20th to the first decade of the 21st century (Hansen et al. 2006), the North-Atlantic has warmed faster than all other ocean basins (Lee et al. 2011). Furthermore, SST isotherms (important delimiters of biogeographic regions), shifted 30–100 km/decade northwards from 1975 to 2005 (Hansen et al. 2006) and the 15°C summer isotherm shifted 330 km northwards from 1985 to 2000 (McMahon and Hays 2006). Under Intergovernmental Panel on Climate Change (IPCC) projections, isotherms will further shift up to 600 km northwards (Hansen et al. 2006) and annual mean SST may increase by 4°C (highest toward the poles) on North-Atlantic rocky shores until the end of the 21st century (Müller et al. 2009). And finally, based on an expected temperature increase of 2°C and observed distributional changes in the English Channel in response to a 0.5°C increase, Southward et al. (1995) suggested that pelagic and benthic communities in the North-Atlantic will shift 300–400 km North.

A global pole-ward shift of temperate seaweed species in response to increasing temperatures is not simply a predication, but a contemporary phenomenon well documented over the last decade. For example, temperate Australian seaweeds retreated 2° latitude poleward over the past half century (Wernberg et al. 2011). Such range shifts of dominant macroalgal species can have a profound impact on the associated rocky shore community. Thus, removal of the canopy-forming fucoid *Hormosira banksii* from intertidal shores in Southern New Zealand turned an intertidal climax community into areas of bare rock with drastically reduced diversity (Lilley and Schiel 2006; Schiel and Lilley 2007, 2011). Algal richness also decreased at two sites in California (Sagarin et al. 1999; Schiel et al. 2004), where foliose algae vanished under a 1–3°C increase in SST and were replaced by more stress-resistant turf-communities and crustose algae (Airoldi 1998; Worm et al. 1999; Connell 2005). Bertocci et al. (2010) found depleted areas of bare rock to be more vulnerable to mechanical disturbance such as human trampling and storms, the latter increasing under climate change (Michener et al. 1997; Easterling et al. 2000).

Given their key role in the intertidal ecosystem and their direct dependence on temperature, seaweeds provide an excellent system in which to investigate the impact of climate change. While we expected that seaweeds will respond to climate change with a poleward shift, few studies have estimated its extent and pattern on a large spatial scale. For example, Müller et al. (2009) predict the poleward shift of mainly subtidal algae (e.g., the kelp species *Laminaria solidungula* and *Saccharina latissima*) in cold-temperate and polar regions on both hemispheres. Similarly, Wernberg et al. (2011) predict a poleward shift of up to 450 km for Australian seaweeds until the end of the 21^st^ century. Martínez et al. (2012b) focused on distributional changes of intertidal macroalgae along the shores of the North-Iberian Peninsula, but the potential northward shift of intertidal macroalgae on a basin-wide scale along temperate North-Atlantic rocky shores is currently not known.

### Predominant macroalgae on North-Atlantic rocky shores

**Figure 1:**
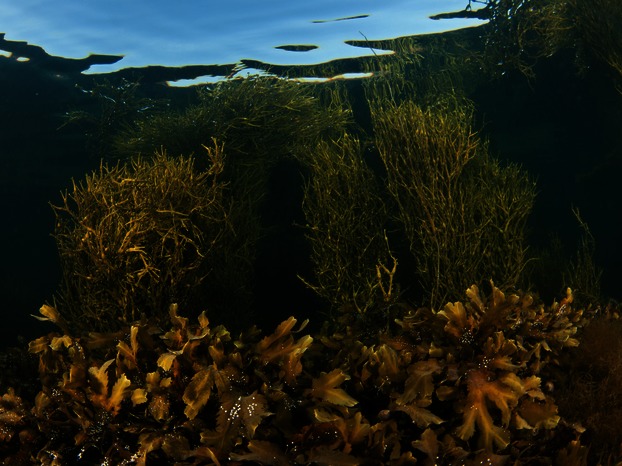
The canopy-forming macroalgae *Ascophyllum nodosum* (top) and *Fucus serratus* (bottom), two of the most predominant foundational key species on temperate North-Atlantic rocky shores (Photo: Galice Hoarau).

We based our investigation on three foundational macroalgal species of North-Atlantic shores (Fig. 1), whose distribution limits coincide with phytogeographic boundaries (Van den Hoek 1975), *Fucus serratus*, *Fucus vesiculosus,* and *Ascophyllum nodosum* (Lüning et al. 1990; Chapman 1995; Wahl et al. 2011). Along the Northeast-Atlantic coast, the three species reach their northern distribution limit at the 10°C summer isotherm (upper limit of the cold-temperate province) in the White Sea with *F*. *vesiculosus* extending south to the Canary Islands (Haroun et al. 2002) (20°C winter isotherm and lower limit of the warm-temperate province) and both *F*. *serratus* and *A*. *nodosum* south to North-Portugal (Arrontes 1993; Araújo et al. 2009; Pearson et al. 2009; Bertocci et al. 2011; Viejo et al. 2011; Martínez et al. 2012b). In the Northwest-Atlantic, *A*. *nodosum* extends from Southern Newfoundland (Canada) to Long Island, NY and *F*. *vesiculosus* extends from Southern Newfoundland (Canada) to Beaufort NC (Adey and Hayek 2005; Keser et al. 2005; Muhlin and Brawley 2009; Olsen et al. 2010). Coastlines further south are mainly sandy and thus uninhabitable for most benthic macroalgae (Van den Hoek 1975). Furthermore, the maximum SST on these shores (28°C) exceeds and thus the lethal limits of *F*. *serratus* (25°C), *F*. *vesiculosus* and *A*. *nodosum* (both 28°C) (Lüning 1984; Lüning et al. 1990; Keser et al. 2005). *Fucus serratus* was introduced to Nova Scotia from Europe at least twice in the late 1860s and has generally expanded its range throughout Nova Scotia, although in an unpredictable manner (Brawley et al. 2009; Johnson et al. 2012). In the central Atlantic, *A*. *nodosum* and *F*. *vesiculosus* occur on Greenland (South and Tittley 1986; Lüning et al. 1990; Muhlin and Brawley 2009) and all three species on Iceland (South and Tittley 1986; Lüning et al. 1990; Kalvas and Kautsky 1998; Ingolfsson 2008), with *F*. *serratus* introduced to Iceland from Southern Norway during the 19th century (Coyer et al. 2006).

### Objectives

Our main objective was to estimate the extent and pattern of northward distribution shifts for intertidal canopy-forming macroalgae on a basin-wide scale along temperate North-Atlantic rocky shores under predicted climate change. We developed correlative Ecological Niche Models for the three seaweed species *F*. *serratus*, *F*. *vesiculosus,* and *A*. *nodosum* under three climate change scenarios for the next 200 years to answer two specific questions: (1) Will the seaweed-based intertidal community shift as an assemblage or as some subset of component species? and (2) Which rocky shores will experience the largest change in their macroalgal composition?

## Materials and Methods

Correlative Ecological Niche Models estimate the ecological niche of a species based on its geographic occurrence and the environmental conditions at the occurrence sites. Projections of the future state of these environmental factors are then used to predict distributional changes of the species in geographic space. We used the program MAXENT v3.3.3e (Phillips et al. 2006; Phillips and Dudík 2008) to trace changes in the geographic distribution of *F*. *vesiculosus*, *F*. *serratus*, and *A*. *nodosum* over the next two centuries. Compared to other niche modeling approaches, MAXENT is one of the programs providing highest predictive performance (Elith et al. 2006).

### Occurrence records

**Figure 2:**
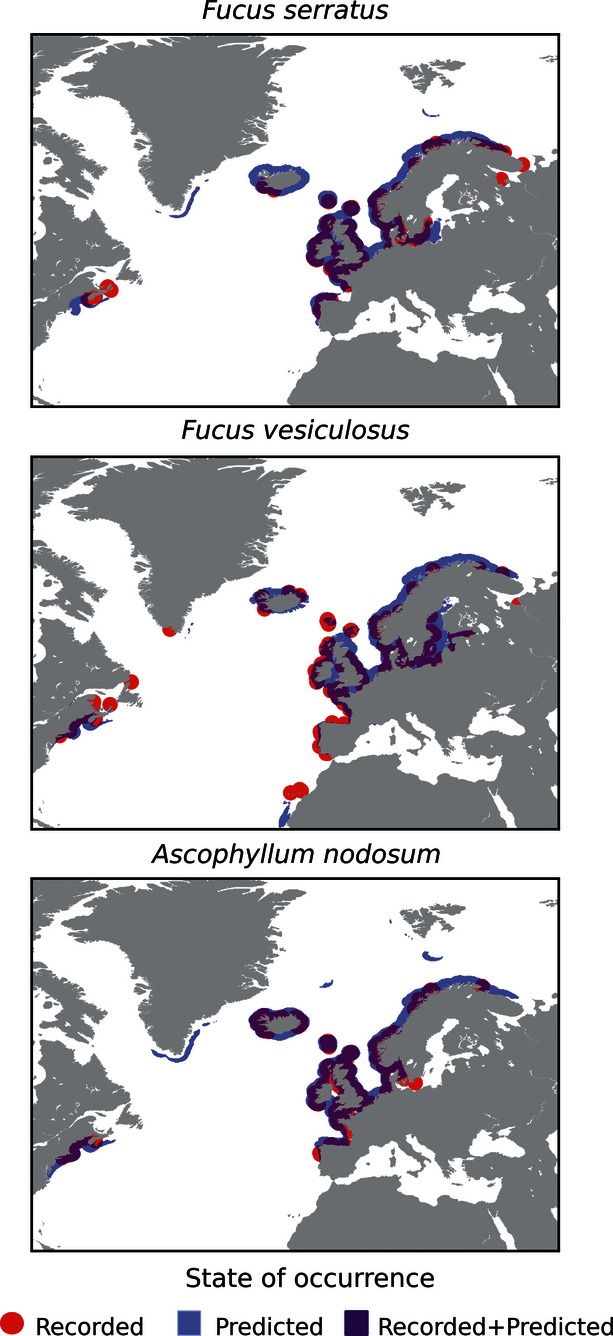
Occurrence records and predicted habitat suitability of the three macroalgal species *Fucus serratus*, *Fucus vesiculosus*, and *Ascophyllum nodosum* under present-day conditions. Suitable versus non-suitable habitat conditions are based on threshold values that best reflected the species' contemporary N and S distribution limits (*F*. *serratus*: 0.4, *F*. *vesiculosus*: 0.4, and *A*. *nodosum*: 0.3). The boundary line at 26°W separates the regions we refer to as West- and East-Atlantic.

For all three species, we utilized three types of occurrence records compiled after 1980: (1) literature, (2) personal observations, and (3) two databases (Appendix S1). Occurrence records, however, can be geographically biased toward easily accessible sites (e.g., coastal roads) and consequently distort the information under which environmental conditions a species thrives best (Phillips et al. 2009). Thus, in order to reduce the possibility that the model overvalues the environmental conditions at these sites and undervalues the environmental conditions in areas of low sampling density, we thinned the set of occurrence records with the Java program “OccurrenceThinner” v.1.01 (Verbruggen 2012b) using thresholds *t*1 = 0.2 and *t*2 = 1.0. Kernel density grids, created with the bkde2D function of the R package “KernSmooth” version 2.23 (Wand 2010) (using a bandwidth of 3.0 in longitudinal and 1.5 in latitudinal direction). We repeated thinning until the sample density showed a smooth distribution lacking high local densities. After bias removal, the data set of *F*. *vesiculosus*, *F*. *serratus,* and *A*. *nodosum* presence records, comprised 115, 130, and 216 locations, respectively (Fig. 2).

### Environmental conditions

The environmental conditions along the North-Atlantic coast, represented by geographic information system (GIS) rasters, were averaged temporally over ≥1 month and spatially at a maximum resolution of 9.2 km × 9.2 km and thus do not accurately reflect the spatial and temporal small-scale variation in the marine intertidal. However, the rasters account for micro-scale fluctuations (e.g., areas of higher average temperatures are likely to also reach higher upper thermal extremes) and their resolution is sufficient for our main aim (Pearson and Dawson 2003): to provide a first approximation of the extent and pattern of range shift for our three focal species on a basin-wide scale.

### Set of present day grids

We considered an initial set of 19 environmental variables of which 15 were represented by GIS rasters of marine environmental conditions at a resolution of 5 arcmin or 9.2 km from Bio-ORACLE, a comprehensive global data set of marine environmental rasters (http://www.oracle.ugent.be/, [Tyberghein et al. 2012]). Since extreme cold or warm air temperatures can be lethal for intertidal species (e.g., Schonbeck and Norton 1978; Firth and Hawkins 2011), we additionally compiled four rasters of surface air temperature (SAT) derivatives: the mean, minimum, maximum, and range (difference between maximum and minimum) of monthly averages over an 8-year period (from January 2003 to December 2010) from remotely sensed daily records (AIRX3STD Level-3 product, version 5) of the Atmospheric Infrared Sounder (AIRS) (http://disc.sci.gsfc.nasa.gov/AIRS/data-holdings), adjusted to a resolution of 1° × 1° using bilinear interpolation with the R package “raster” (Hijmans and van Etten 2011). The rasters of present-day SAT derivatives can be downloaded from http://www.oracle.ugent.be/download.html. To build models of appropriate complexity that were neither under- nor over-fitting, we successively excluded variables from this initial set of 19 environmental rasters in seven steps (see Appendices S2–S4). For the first exclusion step (from Model 1 to Model 2), we used an automatic variable selection procedure implemented in the software MMS v.1.01 (Verbruggen 2012a) that indicates which variables significantly increase or decrease model performance when included in (forward selection), or excluded from (backward selection) the model.

Model performance was based on values of the area under the curve (AUC) of a receiver operating characteristic (ROC) that indicate the ability of the model to discriminate between presence and absence sites (Hanley and McNeil 1982; Fielding and Bell 1997). In Model 2, we retained only those variables giving significant results in both forward and backward selection, and those contributing more than 1% to the regularized gain of the MAXENT model. Subsequently, we successively reduced the model complexity by excluding predictors of lowest contribution to the model until left with a minimum of three environmental variables (see Appendices S2–S4). The relative contribution of these variables to the model gain is listed in Table 1 and their influence on the model prediction is shown in Appendix S5. We then assessed model performance with the program ENMTools (Warren et al. 2010) from MAXENT model raw output grids with all occurrence sites used to train the model and chose for each species the variable set giving highest model-performance (see Appendices S2–S4).

**Table 1:** Contribution of environmental variables to the Ecological Niche Model of each species. Sea surface temperature (SST) derivatives were the most important variables, followed by diffuse attenuation (DA), salinity, and surface air temperature (SAT) derivatives

Variable	Derivative	Unit	Contribution (%)		

			*Fucus serratus*	*Fucus vesiculosus*	*Ascophyllum nodosum*
SST	Minimum	°C	66	46.4	82.3
SST	Maximum	°C	24.7	42.8	
SST	Mean	°C	9.3		
SAT	Minimum	°C			7.3
Salinity	Mean	PSS			10.4
DA	Minimum	m^−1^		10.8	

### Future grids from IPCC scenarios

To project habitat suitability changes over the coming two centuries, we compiled four grids of monthly mean temperature (SST, SAT) derivatives (mean, minimum, maximum, and range) and a grid of average monthly mean salinity conditions over 10-year periods (2087–2096 and 2187–2196) with the R package “raster” (Hijmans and van Etten 2011). These grids represent environmental conditions at the end of the 21st and the 22nd century (from here on referred to 2100 and 2200 conditions), provided by the World Climate Research Programme Coupled Model Intercomparison Project (WCRP CMIP3) multi-model database (http://esg.llnl.gov:8080/index.jsp), and can be downloaded from http://www.oracle.ugent.be/download.html. These future scenarios are based on three IPCC scenarios and represented by the UKMO-HadCM3 model (described in more detail on http://www-pcmdi.llnl.gov/ipcc/model_documentation/ipcc_model_documentation.php and in Gordon et al. (2000); Johns et al. (2003): B1 (550 ppm stabilization), A1B (720 ppm stabilization) and A2 (>800 ppm until 2100). For scenario A2, projections extend only to 2100. We adjusted the resolutions of predicted salinity and SST (1.25° × 1.25° resolution), and predicted SAT (2.75° latitude × 3.75° longitude resolution) to the resolution of the Bio-Oracle grids with the R package “raster” (Hijmans and van Etten 2011), using bilinear interpolation. When predicting future habitat suitability, our models were based on the same variables that we had selected for present-day projections (see Appendices S2–S4). The present-day grids were then replaced with the future grids of the equivalent variables except for “diffuse attenuation” in the model of *F*. *vesiculosus* (see Appendix S3).

### Distribution model choice and settings

For each present-day and future model projection, we performed 10 replicate runs with repeated subsampling of 50% training and 50% test samples from the set of occurrence sites. We ran all models with hinge features only and a regularization parameter β of 0.5, a combination of settings that generally provides models of good performance when there are at least 15 occurrence sites (Phillips and Dudík 2008). To characterize model performance, we calculated average test AUC values over 10 logistic output grids with different random subsamples (50% training and 50% test data) using MAXENT. The AUC value is widely used as an indicator of a model's ability to discriminate between suitable and unsuitable habitat (but see Warren and Seifert 2011 and Jiménez-Valverde 2012 for potential caveats of its use). We converted the logistic model output (averaged over 10 test data sets consisting of random subsamples of 50% of the presence records) to a binary grid that discriminates suitable from non-suitable habitat conditions whereby the clearly identified distribution boundaries of our focal species allowed us to apply fine-tuned thresholds that best reflected the species' contemporary N and S distribution limits: 0.3 for *A*. *nodosum* and 0.4 for both *F*. *serratus* and *F*. *vesiculosus*.

The Ecological Niche Models captured the environmental conditions in the distributional range of the algal species from a set of 10,000 background locations chosen randomly from the North Atlantic coast using the R package “raster” (Hijmans and van Etten 2011). To let MAXENT estimate the environmental limits that separate suitable from non-suitable habitat, we chose background sites from a geographic area that exceeded the realized distribution by a maximum of 15° in both latitudinal and longitudinal direction. We compiled one set of background locations for *F*. *serratus* and *A*. *nodosum*, located within 35° to 80° latitude and −80° to 40° longitude, excluding the Mediterranean and the Black Sea. For the species with the widest distribution range, *F*. *vesiculosus*, the areas of background sites were located within 22° to 85° latitude and −76° to 44° longitude, excluding the Black and Red Sea. We retained the Mediterranean area, as we retrieved two occurrence records for *F*. *vesiculosus* from both the IOBIS and the GBIF databases. Because one of them was recorded in 1848 (we included only records collected after 1980) and we could not confirm if the second record from 2008 was a drift or attached individual, we omitted both from the data set of actual occurrence sites. Nevertheless, these records indicate that the Mediterranean might belong to the potential niche of this species.

### Changes in latitudinal boundaries and length of suitable coastline

For each species, we calculated the overall mean projected latitude of northern and southern distribution boundaries along the West- (≤35° West) and East-Atlantic (≥35° West) coast over all applied scenarios. For present-day projections, the value was based on a single latitude estimate extracted with the R package “raster” (Hijmans and van Etten 2011) from the binary MAXENT output grid of habitat suitability based on the species-specific logistic threshold values. For future projections, it was based on latitude estimates under each IPCC scenario (B1, A1B, and A2 for 2100, B1 and A1B for 2200). From here on, we refer to the present-day predictions as year 2000, although they are based on environmental conditions recorded mainly in the second half of the 20th century and the first decade of the 21st century.

## Results

### Projected present-day niches

In general, the niche projections mirrored the realized distributions (see Fig. 2) although some disagreement with the observed occurrences was apparent. The highest deviation between projected and realized niche of the three species was found for *F*. *vesiculosus* (main discrepancies along the entire West-Atlantic coast and the coast of Africa in the East-Atlantic, see Fig. 2). Accordingly, its model performance (indicated by the test AUC value; the closer to 1, the better the fit of the model to a species' realized niche) was lower compared with that of the other two focal species: 0.86 for *F*. *vesiculosus*, 0.93 for *F*. *serratus*, and 0.93 for *A*. *nodosum*. These are average values of 10 test AUC values that differed in the set of 50% randomly selected test occurrence sites. The AUC value does not specify the models' performance to predict a species' potential niche (Jiménez-Valverde 2012).

#### Northeast-Atlantic

**Figure 3:**
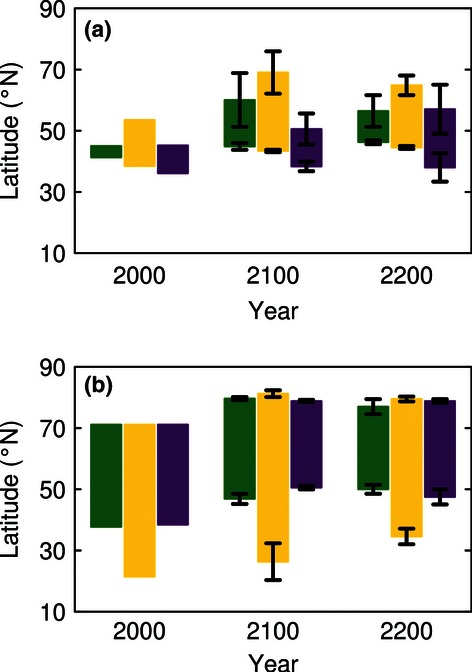
Latitudinal distribution boundaries (°N) for the three algal species (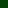 *F*. *serratus*, 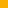 *Fucus vesiculosus*, 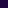 *Ascophyllum nodosum*) in 2000, 2100, and 2200 in the (A) West Atlantic (40°W to 26°W) and (B) East Atlantic (26°W to 50°E), derived from the niche model projections. Bars cover the latitudinal range of suitable habitat conditions. Bars of 1 standard error indicate the variation that is due to disagreements between the Intergovernmental Panel on Climate Change (IPCC) scenarios B1 and A1B for year 2200, and additionally scenario A2 for year 2100. Error bars are missing from the present-day estimates since they are based on a single model projection.

The present southern boundary of both *F*. *serratus* and *A*. *nodosum* is located at ca. 40°N (fitting Northern Portugal), but was projected 390 km and 350 km further south at ca. 38°N and 38.5°N, respectively (Fig. 3B). The projected southern limit of *F*. *vesiculosus* (27°N) was 5.5° latitude (ca. 780 km) further south than the southernmost record of this species on the Canary Islands. This resulted from the minimum SST response curve (Appendix S2) that projected habitat suitability to decrease from 10°C to ca. 17°C and thus to be low along the West-African coast above ca. 21.5° latitude, but to increase and remain suitable at minimum SST values exceeding 17°C (clamping effect), which is reached at the projected southernmost latitude at 21.5° latitude. Even further south, minimum SST values remained suitable but maximum SST values were too high.

The northern boundary of all three species was projected at its actual location (ca. 71°N) in Northern Norway. Although both *F*. *serratus* and *F*. *vesiculosus* occur in the White Sea (Fig. 2), the present-day projection excluded areas further east than Lumbovski Bay at 40°E along the Russian Barents Sea (Fig. 2).

#### Northwest-Atlantic

The southern distribution boundaries for *F*. *serratus* and *A*. *nodosum* are projected 280 km and 540 km too far south, respectively. While *F*. *serratus* occurs only north of Yarmouth Nova Scotia (Canada) at ca. 43°N, and *A*. *nodosum* north of Long Island NY at ca. 40°N, the predicted southern boundaries were ca. 41°N and 36°N, respectively. The southern limit of *F*. *vesiculosus*, which occurs south to Beaufort NC at ca. 34°N, is projected too far north at ca. 38°N (Fig. 3a) and 2° latitude (ca. 280 km) further south than the southernmost occurrence record at ca. 40°N (Fig. 2).

**Figure 4:**
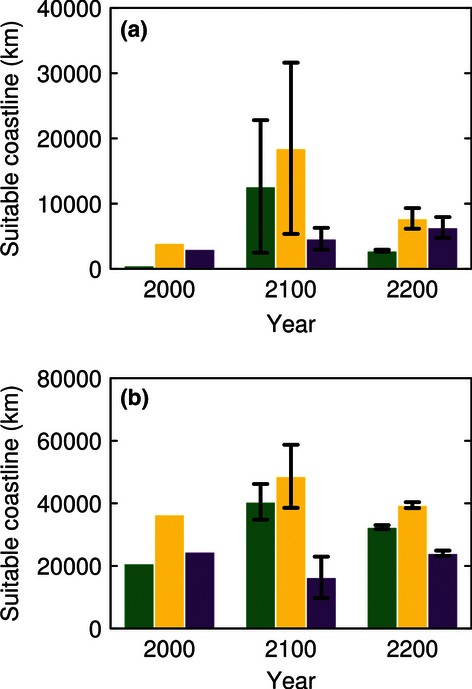
Changes in the length of coastline (in km) with suitable habitat conditions for the three algal species (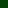 *F*. *serratus*, 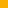 *Fucus vesiculosus*, 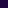 *Ascophyllum nodosum*) from 2000 to 2200 in the (A) West Atlantic (40°W to 26°W) and (B) East Atlantic (26°W to 50°E), derived from the niche model projections. Bars of 1 standard error indicate the variation due to disagreements between the Intergovernmental Panel on Climate Change (IPCC) scenarios B1 and A1B for year 2200, and additionally scenario A2 for year 2100. Error bars are missing from the present-day estimates as they are based on a single model projection.

The projected northern limit of *F*. *serratus*, at ca. 45°N (Fig. 3A), closely matched its actual northern boundary in Nova Scotia (ca. 140 km further north at 46°N, Fig. 2). The northern projection for *A*. *nodosum* also was ca. 45°N (Fig. 3A), only 9 km south of its northernmost occurrence record (Fig. 2). The projected northern boundary of *F*. *vesiculosus* at ca. 53.5°N matched its northernmost occurrence record in North-Canada well (see Fig. 2). It is important to realize that offshore areas where habitat is predicted to be suitable, were excluded from the estimations of the length of habitable coastline (Fig. 4A, B) and the latitudinal range boundaries (Fig. 3A, B), since the seaweeds are only able to track suitable habitat directly along the shore. For example, the coast of Greenland was regarded as unsuitable habitat for any of the three species in Fig. 2, although the model projected suitable habitat several km off Greenland's coast.

### Predicted niche shifts

The climate change projections contained novel climate conditions in the southern ranges of the species' distribution with temperatures exceeding the maximum values of both, occurrence records and background samples. This was indicated by negative values (data not shown) in the multivariate similarity surfaces (geographic rasters provided by MAXENT that show for each raster pixel how similar the predicted environmental conditions are to present-day conditions; Elith et al. 2010), generally south of Spain in the East-Atlantic and south of Cape Cod MA on the West-Atlantic coast. For the A2 scenario projections, novel climate conditions extended to the United Kingdom on the East- and Nova Scotia on the West-Atlantic coast. The most dissimilar variables (MoD) between present and future conditions were minimum SST for all three species and minimum SAT in addition for *A*. *nodosum*. We allowed MAXENT to “clamp” values that exceeded the training range by setting them to the maximum value captured by training samples, so that the response remained constant and equal to the upper limit of the training range. We assumed that the projected loss of suitability in these areas was still correct, since the background samples captured the species' upper temperature limits during training, so that minimum SST and SAT approached a prediction of zero near the upper limit before clamping had an effect on the models of *A*. *nodosum* and *F*. *serratus* (Appendix S2). For *F*. *vesiculosus* however, minimum SST values at the upper training range were still within the species tolerance range and thus the models projected minimum SST values to remain suitable even though they might rise beyond the upper tolerance limits (Appendix S2), resulting in future model projections that might underestimate the future habitat loss. “Clamping” was not necessary for projections into the Arctic areas.

### Habitat loss

**Figure 5:**
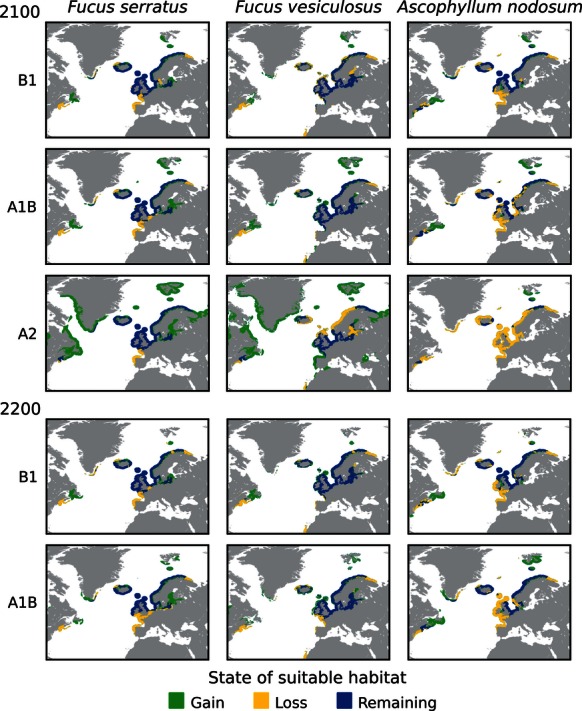
Habitat suitability changes of the three algal species *Fucus serratus*, *Fucus vesiculosus*, and *Ascophyllum nodosum* in the North-Atlantic over the coming two centuries under the Intergovernmental Panel on Climate Change (IPCC) scenarios B1, A1B, and A2. Suitable versus non-suitable habitat conditions are based on threshold values that best reflected the species' contemporary N and S distribution limits (*F*. *serratus*: 0.4, *F*. *vesiculosus*: 0.4, and *A*. *nodosum*: 0.3). The boundary line at 26°W separates the regions we refer to as West- and East-Atlantic.

All climate change scenarios, including the weakest (B1), predicted habitat loss for the three target species along their present southern distribution limits by 2100 (Fig. 5). The average northward retreat of all species is predicted to be more pronounced on the East- (2100: 8.7 ° latitude N, 2200: 11.5° latitude N) compared to the West-Atlantic coast (2100: 3.6° latitude N, 2200: 4.3° latitude N). It should be noted that the predicted habitat loss is on the conservative side, since almost all models overestimated the present-day distribution toward the south, a bias that is likely to transfer to the future projections. The predicted habitat loss thus includes the areas that are very likely to turn into seaweed-depleted barren grounds and will potentially prove to be even more extreme.

#### Northeast-Atlantic

On the Northeast-Atlantic coast, suitable habitat for *F*. *serratus* and *A*. *nodosum* is projected to retreat at least as far north as Brittany in France. The southern boundary shifted further north for *F*. *serratus* (ca. 1370 km to 50°N) than for *A*. *nodosum* (ca. 1010 km to 47.5°N) until 2200 (Fig. 3B). *Fucus vesiculosus* may lose most habitat along the Atlantic coast of Africa, Spain and Portugal until 2200 (ca. 1460 km northward shift, see Fig. 5). While it may retreat from present occurrences in the Canary Islands and off the West-African coast, habitat may remain locally suitable south to ca. 35°N (Fig. 3B). Moreover, the B1 and the A1B scenarios consistently predicted the Russian Barents Sea coast to become too cold to sustain populations of any of the three algal species (Fig. 5).

#### Northwest-Atlantic

On the Northwest-Atlantic coast, the B1 and A1B scenarios predicted shores south of Halifax in Nova Scotia (ca. 45°N, Fig. 3A), to become uninhabitable by 2200 for both *F*. *serratus* (ca. 550 km northward shift) and *F*. *vesiculosus* (ca. 680 km northward shift, Fig. 5). In contrast, the southern distribution limit of *A*. *nodosum* remained at ca. 38°N (210 km northward shift on average, Fig. 3A) and the B1 scenario predicted a gain of suitable coastline south of its present-day distribution (Fig. 5).

### Habitat gain

The distribution models predicted habitat gain in the north for all three species. The total suitable habitat is predicted to increase on almost every coast since habitat gain in the north exceeded habitat loss in the south except for *A*. *nodosum* on the Northeast-Atlantic coast (see Figs. 4A, B, and 5).

#### Northeast-Atlantic

Southern Spitsbergen is projected as suitable habitat for all three species by 2100. The B1 and A1B scenarios predicted habitat loss along the Russian Barents Sea coast. In contrast, the A2 scenario predicted up to 10°C higher SST and thus suitable conditions east of the White Sea coast for *F*. *serratus* and *F*. *vesiculosus* by 2100 (Fig. 5).

#### Northwest-Atlantic

In the Northwest-Atlantic, the A1B scenario predicted appropriate habitats for all three species in Newfoundland and the southern parts of Greenland by 2200. The A2 scenario predicted almost the entire Northwest-Atlantic coast of Canada and Greenland as suitable habitat for *F*. *serratus* and *F*. *vesiculosus* (Fig. 5), explaining the high average gain of suitable coastline with wide error bars and the far northward shift of the average latitudinal distribution boundary by 2100 (Figs. 3A and 4A).

### Stable coastlines

The models projected almost no present-day suitable habitat in the Northwest-Atlantic to remain suitable for all three species over the next two centuries (Figs. 3A and 5). In contrast, the Northeast-Atlantic coastline from ca. 70°N in Northern Norway to 50°N in South-England likely provides consistently suitable habitat for all three species (Fig. 3B) and thus will encounter least ecological changes.

## Discussion

### Where climate change will have the highest impact

The main objective of our study was to investigate the impact of climate change on the distribution of canopy-forming seaweeds along North-Atlantic rocky shores. While a poleward shift of seaweed communities might be an expected response to climate change, our study makes two major contributions in specifying the extent and pattern of shift explicitly.

The first main finding of our study is that our focal seaweed species will shift northwards as an assemblage. Although we have treated our three focal species as separate units, their predicted relative distribution in 2200 closely resembled the distribution pattern in 2000 (Fig. 3A, B). For example, in the East-Atlantic the northern limits of the three species were close to each other and the southern limit of *F*. *vesiculosus* reached furthest south (Fig. 3B) in 2000 and 2200. With an assemblage-like northward shift of the temperate macroalgal flora, warm-temperate shores will lose their key foundational species while species-rich seaweed communities are likely to establish in polar areas.

The second main contribution of our study is the identification of North-Atlantic rocky shores that will experience the largest change in their macroalgal composition: (1) the warm-temperate East-Atlantic region from Portugal up to Brittany, France, where *F*. *serratus* and *A*. *nodosum* (this study) and other species such as *S. latissima*, *Laminaria hyperborea,* and *Chondrus crispus* (Müller et al. 2009) are predicted to become extinct; (2) the Southern Arctic region, including Northern Canada, Greenland, and Spitsbergen, into which temperate species may immigrate; (3) the Northwest-African shore on which *F*. *vesiculosus* will markedly decline; and (4) the Northwest-Atlantic coast of the United States, where only *A*. *nodosum* is predicted to persist. These last two coastlines are likely to transform into entirely different systems because canopy-forming seaweed species are absent from the adjacent sandy shores and the marine flora in the more southern tropical West-Atlantic differs markedly from the cold-temperate region (Van den Hoek 1975; Michanek 1979).

This study predicts the potential northward shift of intertidal canopy-forming macroalgae along temperate North-Atlantic rocky shores for the first time on a basin-wide scale. The predicted northward shift in the West-Atlantic (3.6° latitude N until 2100) complies with the predictions of Wernberg et al. (2011) for temperate Australian seaweeds (1.7° to 5° latitude until 2070). The shift on the East-Atlantic coast is predicted to be higher (8.7° latitude N on the East-Atlantic coast).

These predictions are insensitive to potential climate change refugia that could result from the small-scale variability of SAT (Hampe and Petit 2005; Austin and Van Niel 2011; Seabra et al. 2011; Martínez et al. 2012b), since our habitat models were mainly based on the more homogeneous SST (Seabra et al. 2011). Moreover, at a resolution of 9.2 km^2^, our models captured the scale of SST variability at which thermal refugia occurred (see for e.g., *Alaria esculenta* on the south-coast of the UK, Hiscock et al. 2004; Müller et al. 2010). The only potential cold-thermal refugia our models may have missed are cool water masses that reach shallow depths in Northeast-Canadian fjords and are inhabited by the Arctic kelp *L. solidungula*, for which the adjacent open shore temperatures are too warm (reviewed in Müller et al. 2009, 2010).

The general agreement of our models with the occurrence records of the three fucoid species (see Fig. 2) supports the view that climatic factors (mainly SST derivatives in our case; see Table 1) are sufficient to provide a first approximation of niche shifts under climate warming (Breeman 1990; Huntley et al. 1995; Pearson and Dawson 2003; Araújo and Guisan 2006). However, to what extent our predicted niche shift will be realized depends on intrinsic characteristics of the investigated species as well as extrinsic biotic and abiotic factors.

## Loss at the southern rear edge

The predicted habitat loss along the species' southern rear edges will have a profound impact on the associated rocky shore community. For example, removal of the canopy-forming fucoid *H. banksii* from intertidal shores in southern New Zealand, caused the loss of other fucoid and coralline algae, increased the area of bare rock up to tenfold and reduced the diversity in the associated community by up to 44% (Lilley and Schiel 2006; Schiel and Lilley 2007, 2011). Sagarin et al. (1999) and Schiel et al. (2004) found that such ecosystem shifts from shores dominated by canopy-forming macroalgae to communities of turf forming algae and barren grounds with large areas of bare rock can result from the direct negative impact of rising SST on canopy-forming and foliose intertidal algae.

### Empirical evidence

The direct negative impact of climate change on the southern edge populations of our focal species is not only a prediction but is already supported by empirical findings. For example, on the Northeast-Atlantic coast, the abundance of *F*. *serratus* decreased by over 90% during the last decade off Ribadeo (Northern Spain) (A. Jueterbock, and J. Coyer, pers. obs., see Appendix S6), presumably due to SST routinely reaching lethally high levels (>22°C) (Martínez et al. 2012a). Besides having low genetic diversity (Coyer et al. 2003), the present southern edge populations of *F*. *serratus* are likely to thermal stress (Pearson et al. 2009), and have recently declined in reproductive capacity and minimum size of reproduction (Viejo et al. 2011).

The southern edge populations of *A*. *nodosum* also suffered enhanced mortality and invested increasingly in reproductive output at the expense of growth (Araújo et al. 2011) on the Northeast-Atlantic coast. On the Northwest-Atlantic coast, the abundance of *A*. *nodosum* decreased from Nova Scotia and New Brunswick (Canada) *F*. *vesiculosus*, putatively because of increasing water and air temperatures (Ugarte et al. 2009). Thus, the northward retreat of *A*. *nodosum* from Long Island and further north may be even more extensive than that our models predict (see Fig. 3A).

### Increasing grazing pressure

The predicted northward shift could be accelerated by the indirect effect of elevated SST to increase herbivore abundance and activity on Northeast-Atlantic shores from high to low latitudes (Thompson et al. 2000; Jenkins et al. 2001; Hawkins et al. 2008). While being currently highest in the southern-most portions of the seaweeds' distribution range, grazing pressure progressively increases northward under climate change (Southward et al. 1995; Davies et al. 2007; Hawkins et al. 2008). By reducing recruitment (Jenkins et al. 1999; Cervin et al. 2005; Jonsson et al. 2006; Hawkins et al. 2008) and growth rate (Toth et al. 2007), grazing directly decreases the abundance of fucoids directly (Jenkins et al. 2005; Davies et al. 2007; Lorenzen 2007). With a generation time of 1–2 years (e.g., Coyer et al. 2007), *F*. *serratus* and *F*. *vesiculosus* depend on nearly annual germling recruitment and thus are putatively more susceptible to the increase of microphagous grazing activity than *A*. *nodosum* with a generation time of 50–70 years (Olsen et al. 2010). However, limpets were also found to entirely graze down mature *A*. *nodosum* monocultures (Lorenzen 2007). Furthermore, the experimental removal of *A*. *nodosum* from the Isle of Man resulted in a threefold–sixfold increase in limpet density, which both prevented algal recruitment and increased the area of bare rock by 49% in the following 12 years (Jenkins et al. 1999, 2004). Thus, an initial decrease in algal abundance through thermal stress can trigger a positive feedback loop through which increasing dominance of herbivorous grazers further reduces algal recruitment and ultimately, causes the disappearance of entire seaweed beds.

### Plastic and adaptive responses

Despite the empirical data showing that southern habitat loss of our focal canopy-forming seaweeds has already started, there remains an uncertainty that generally limits the predictability of correlative bioclimate envelope models: the species' intrinsic potential to adapt to the thermal shift through phenotypic plasticity or evolutionary adaptation (Pearson and Dawson 2003; Thuiller et al. 2008; Lavergne et al. 2010). Such plastic or adaptive capacities might mitigate the predicted retreat of the seaweeds' rear-edges, as these represent ancient glacial refugia in which the species survived the Last Glacial Maximum (LGM), 18 to 20 kya (reviewed in Maggs et al. 2008; Provan and Bennett 2008). Specifically, three refugia are recognized: (1) The Brittany region (e.g., Hurd Deep) for all three species (Hoarau et al. 2007; Olsen et al. 2010; Coyer et al. 2011b); (2) Southwest-Ireland for the two *Fucus* species (Coyer et al. 2003, [Bibr b36][Bibr b37]; Hoarau et al. 2007); and (3) the Northwest-coast of the Iberian Peninsula for *F*. *serratus* (Coyer et al. 2003; Hoarau et al. 2007). Due to their long-term persistence, southern-edge populations are generally centers of genetic diversity with unique alleles (Hampe and Petit 2005; Maggs et al. 2008; Diekmann and Serrao 2012), and played an important role for species persistence and taxa diversification throughout the Quaternary (Hewitt 1996; Hampe and Petit 2005). Populations at the southern edge of a northward moving species usually become extinct (Aitken et al. 2008), thereby reducing standing variation, biodiversity, and adaptive potential of the species on a massive scale (Bálint et al. 2011; Bijlsma and Loeschcke 2012; Provan and Maggs 2012). On the North-Iberian Peninsula for example, despite being an ancient glacial refugium for *F*. *serratus*, genetic diversity was reduced during recurrent cycles of thermally induced extinctions and recolonizations (Arrontes 1993, 2002; Coyer et al. 2003). Whether the southern-edge populations will become extinct or if they can mitigate the predicted northward shift is an open question of crucial importance for the entire North-Atlantic rocky shore ecosystem, but patterns of local adaptation and the adaptation potential of our focal species are too poorly understood to know whether they could mitigate the predicted northward shift.

## Expansion of the northern leading edge

While southern temperate regions are becoming too warm, sub-Arctic and Arctic coastal areas along Southern Greenland and Spitsbergen are predicted to provide suitable habitat for the fucoid seaweeds in the coming two centuries (Figs. 3A, B and 5). The northward expansion of the seaweeds' leading edge is afflicted with a much higher uncertainty than the predicted habitat loss along their southern rear edge, since the factors that mediate successful colonization of Arctic regions are poorly understood. Our study makes the first step in predicting where the focal species are potentially able to establish new colonies, but we are unable to predict if, where, or how rapid they will colonize the potentially suitable Arctic rocky shores in the next 200 years. Whether or not our focal seaweed species can track the predicted pole-ward shift to isolated Arctic shores will depend on the following three main factors.

### Dispersal and invasive potential

As fucoid zygotes generally settle <10 m from the egg-bearing female (Arrontes 1993, 2002; Serrão et al. 1997; Dudgeon et al. 2001), long-range dispersal must involve drifting thalli of reproductively mature individuals. Both *F*. *vesiculosus* and *A*. *nodosum* bear air vesicles that allow flotation of thalli in surface waters and consequently, are more likely to drift to distant shores (John 1974; Van den Hoek 1987; and citations therein) than *F*. *serratus*, which lacks flotation vesicles and sinks if not attached to flotsam or jetsam. The inability of *F*. *serratus* to disperse via floating thalli is reflected by a small panmictic unit of 0.5–2 km (Coyer et al. 2003, 2011a) and a slow natural dispersal rate of 0.2–0.6 km/year (Coyer et al. 2006; Brawley et al. 2009). Shipping traffic, which can generally increase algal dispersal rates by an order of magnitude (Lyons and Scheibling 2009), may account for the more recent estimate of 2.6 km/year (up to 11 km/year) for *F*. *serratus* along Northwest-Atlantic shores (Johnson et al. 2012). Although modern ships use water instead of rocks as ballast, they still can facilitate dispersal of macroalgae through hull-fouling, accidental entanglement in anchors or fishing gear, or deliberate use as packing material (Hewitt et al. 2007; Lyons and Scheibling 2009; Johnson et al. 2012). Shipping transport has increased in the Canadian and Russian Arctic (Lasserre and Pelletier 2011) in response to loss of Arctic sea-ice (Serreze et al. 2007) and undoubtedly will play an important role in the introduction of marine species into polar areas (e.g., Clayton et al. 1997; Brawley et al. 2009; Johnson et al. 2012).

Because shipping facilitates transport of clusters of individuals, it might also overcome the requirement of dioecious species to have at least one individual of each sex settling close enough for successful sexual reproduction. For example, the relatively poorly dispersing *F*. *serratus* colonized shores of North America, Iceland and the Faroe Islands through human shipping (Coyer et al. 2006; Brawley et al. 2009; Johnson et al. 2012). In contrast, shipping activities may be unimportant to *A*. *nodosum* or *F*. *vesiculosus*. The former species is a good disperser, but its long generation time of 50–70 years (Olsen et al. 2010), slow growth, and high early post-settlement mortality of recruits (Jenkins et al. 1999, 2004) may prevent tracking the predicted northward shift. The latter species has expanded 154 km (average rate of 3 km/year) southwards along the Portuguese coast in the past 50 years (Lima et al. 2007) and conceivably could disperse up to 600 km along suitable coastline within the next two centuries, even without shipping activities.

### Critical day length and polar night

Photoperiod, along with temperature, regulates seaweed reproduction (Dring and Brown 1982; Santelices 1990; Brawley and Johnson 1992; and references therein). For example, *A*. *nodosum* and *F*. *vesiculosus* start producing receptacles after the autumn equinox when the day length reaches a critical value of 12 h (Terry and Moss 1980; Bäck et al. 1991; but see Berger et al. 2001). As correlative habitat models do not extrapolate the co-variation between day length and temperature to the future, they cannot reliably predict the presence of a seasonal window during which critical levels of photoperiod and temperature coincide in polar areas. However, the presence of *A*. *nodosum* and *F*. *vesiculosus* on sub-Arctic shores of Southern Greenland and their plasticity in phenology (e.g., Brawley and Johnson 1992) suggests that they can optimize reproduction on shores along Greenland and Svalbard (where all of the three focal species were enlisted as present in South and Tittley (1986)). Of equal importance, however, might be the ability of the focal species to tolerate the nearly 4-month polar night on Svalbard, a dark period that lasts nearly two times as long as at their present northern distribution limit in Northern Norway (Lüning et al. 1990). A key question is whether they can store photosynthetates and nitrogen reserves as can the cold-temperate kelp species *Laminaria hyperborea* (Lüning et al. 1990; and references therein), which recently colonized shores along Southern Svalbard (Peltikhina, 2002; Olsen et al., 2004; quoted in Müller et al. 2009, 2010). The increase in nitrogen tissue concentrations in *A*. *nodosum* and *F*. *vesiculosus* after the growing season in autumn (Asare and Harlin 1983; Chopin et al. 1996) might indicate that these species are able to store nitrogen. Moreover, mannitol, a compound for reserve storage of photosynthetates (Bidwell and Ghosh 1962; Bidwell 1967), occurs in all three focal species in osmotically relevant concentrations (Reed et al. 1985), and might allow them to survive and grow during long dark periods in the Arctic (Lehvo et al. 2001).

### Competitive interactions

As polar algae are mainly restricted to the subtidal zone (Wiencke and Amsler 2012), competitive interactions likely will be minimal in the intertidal. The dominant algal species in the Arctic intertidal is *F. distichus* (Lüning et al. 1990; Wiencke and Amsler 2012), which is unlikely to prevent colonization of southern species during climate change. For example, *F*. *serratus* replaced *F*. *distichus* in the lower intertidal after the former's introduction to Iceland (Ingolfsson 2008). Furthermore, *F*. *serratus* recruited within dense algal canopies (Arrontes 2002) and out-competed *F*. *distichus* and other seaweeds from intertidal and shallow subtidal shores after it had colonized Nova Scotia (Johnson et al. 2012). Negative competitive interactions between *F*. *distichus* and either *A*. *nodosum* or *F*. *vesiculosus*, however, are not apparent as all three co-occur on the same shore at slightly different zonation levels (Ellis and Wilce 1961; Munda 2004).

## Conclusion

Our Niche Models predict that the predominant foundational macroalgae of the North-Atlantic rocky intertidal will shift northwards as an assemblage and by 2100 will have lost most of their habitat south of 45°N, while suitable environments are opening up in the Arctic. Empirical findings provide strong support for that the areas we predict to become unsuitable will indeed turn into barren grounds without canopy-forming seaweeds. A remaining key question is, whether the plastic or adaptive capacities of southern-edge populations in ancient glacial refugia are sufficient to survive climate change or if these centers of unique genetic diversity will become extinct. If or where the temperate seaweeds will colonize the Arctic rocky shores, which we predict to become suitable in the next 200 years remains unclear as seaweed dispersal, dark tolerance, and competitive interactions in the Arctic intertidal are poorly understood.

## Supporting Information

**Appendix S1.** Literature records and data sets accessed through the GBIF and OBIS databases.

**Appendix S2**. Environmental variable selection for *Fucus serratus*. The table shows the positive effect of decreasing Niche Model complexity (included environmental rasters are marked with an x) on model performance, which is based on the two high-performance model selection criteria (Warren and Seifert 2011): (1) sample size corrected Akaike information criterion (AICc) (Akaike 1974), and (2) Bayesian information criterion (BIC) (Schwarz 1978). AICc/BIC values could not be calculated where the number of model parameters exceeded the number of occurrence sites. The environmental rasters included in the model of the highest performance (model 8, marked in bold) were selected for modeling the niche of *F. serratus* under present-day and future conditions and their model-contribution is listed in Table 1. DA, diffuse attenuation; Dissox, dissolved oxygen; PAR, photosynthetically active radiation; SAT, surface air temperature; SST, sea surface temperature.

**Appendix S3**. Environmental variable selection for *Fucus vesiculosus*. The table shows the positive effect of decreasing Niche Model complexity (included environmental rasters are marked with an x) on model performance, which is based on the two high-performance model selection criteria (Warren and Seifert 2011): (1) sample size corrected Akaike information criterion (AICc) (Akaike 1974), and (2) Bayesian information criterion (BIC) (Schwarz 1978). AICc/BIC values could not be calculated where the number of model parameters exceeded the number of occurrence sites. The environmental rasters included in the model of highest performance (model 8, marked in bold) were selected for modeling the niche of *F*. *vesiculosus* under present-day and future conditions and their model-contribution is listed in Table 1. DA: diffuse attenuation, Dissox: dissolved oxygen, PAR: photosynthetically active radiation, SAT: surface air temperature, SST: sea surface temperature.

**Appendix S4**. Environmental variable selection for *Ascophyllum nodosum*. The table shows the positive effect of decreasing Niche Model complexity (included environmental rasters are marked with an x) on model performance, which is based on the two high-performance model selection criteria (Warren and Seifert 2011): (1) sample size corrected Akaike AICc (Akaike 1974), and (2) Bayesian information criterion (BIC) (Schwarz 1978). The environmental rasters included in the model of highest performance (model 8, marked in bold) were used for modeling the niche of *A*. *nodosum* under present-day and future conditions and their model-contribution is listed in Table 1. DA, diffuse attenuation; Dissox, dissolved oxygen; PAR, photosynthetically active radiation; SAT, surface air temperature; SST, sea surface temperature.

**Appendix S5.** Response curves. Response curves showing the mean change in logistic prediction over ten replicate models in red and the range of two standard deviations as blue shade. They show MAXENT models that were built only with the respective environmental variable and represent how the predicted habitat suitability depends on each variable and on dependencies induced by their correlations with other variables. DA, Diffuse attenuation; SAT, Surface air temperature; SST, Sea surface temperature.

**Appendix S6.** *Fucus serratus* abundance decline. *Fucus serratus* coverage captured in (A) 1999 and (B) 2010 at exactly the same site in Ribadeo (Northwest-coast of Spain; indicated as a red dot in the map inset in [B]). We estimated an abundance decline of ≥90% over this 11-year period.
