# Supplementary material for: Climate change impact on seaweed meadow distribution in the North Atlantic rocky intertidal
Source: Ecol Evol. 2013 Apr 12;3(5):1356–73. doi: 10.1002/ece3.541 (PMC3678489; doi:10.1002/ece3.541)
Supplement: Supplementary file 1 [file ece30003-1356-SD1.pdf]

Literature references

1. ***Fucus vesiculosus***

Bäck S & Ruuskanen A (2000). Distribution and maximum growth depth of *Fucus vesiculosus* along the Gulf of Finland. *Marine Biology*, **136**, 303–307.

Eriksson BK & Johansson G (2003). Sedimentation reduces recruitment success of *Fucus vesiculosus* (Phaeophyceae) in the Baltic Sea. *European Journal of Phycology*, **38**, 217–222.

Germain P, Leclerc G, & Simon S (1995). Transfer of polonium-210 in *Mytilus edulis* (L.) and *Fucus vesiculosus* (L.) from the Baie de Seine (Channel coast of France). *Science of the Total Environment*, **164**, 109–123.

Guillaumont B, Callens L, & Dion P (1993). Spatial distribution and quantification of *Fucus* species and *Ascophyllum nodosum* beds in intertidal zones using spot imagery. *Hydrobiologia*, **261**, 297–305.

Haroun RJ, Gil-Rodríguez MC, Castro JD, & Reine WFP (2002). A checklist of the marine plants from the Canary Islands (Central Eastern Atlantic Ocean). *Botanica Marina*, **45**, 139–169.

Malavenda SV & Voskoboinikov GM (2009). Influence of abiotic factors on the structure of the population of the brown alga *Fucus vesiculosus* in East Murman (Barents Sea). *Russian Journal of Marine Biology*, **35**, 132–137.

Malm T & Kautsky L (2003). Differences in life-history characteristics are consistent with the vertical distribution pattern of *Fucus serratus* and *Fucus vesiculosus* (Fucales, Phaeophyceae) in the central Baltic Sea. *Journal of Phycology*, **39**, 880–887.

Perrin C, Daguin C, Van De Vliet M, Engel CR, Pearson GA, & Serrao EA (2007). Implications of mating system for genetic diversity of sister algal species: *Fucus spiralis* and *Fucus vesiculosus* (Heterokontophyta, Phaeophyceae). *European Journal of Phycology*, **42**, 219–230.

Ruuskanen A, Back S, & Reitalu T (1999). A comparison of two cartographic exposure methods using *Fucus vesiculosus* as an indicator. *Marine Biology*, **134**, 139–145.

Serrão EA, Kautsky L, & Brawley SH (1996). Distributional success of the marine seaweed *Fucus vesiculosus* L. in the brackish Baltic Sea correlates with osmotic capabilities of Baltic gametes. *Oecologia*, **107**, 1–12.

Torn K, Krause-Jensen D, & Martin G (2006). Present and past depth distribution of bladderwrack (*Fucus vesiculosus*) in the Baltic Sea. *Aquatic Botany*, **84**, 53–62.

2. ***Fucus serratus***

Arrontes J (2002). Mechanisms of range expansion in the intertidal brown alga *Fucus serratus* in northern Spain. *Marine Biology*, **141**, 1059–1067.

Arrontes J (1993). The nature of the distributional boundary of *Fucus serratus* on the north shore of Spain. *Marine Ecology Progress Series*, **93**, 183–193.

Brawley SH *et al.* (2009). Historical invasions of the intertidal zone of Atlantic North America associated with distinctive patterns of trade and emigration. *Proceedings of the National Academy of Sciences*, **106**, 8239–8244.

Coyer JA *et al.* (2011). Genomic scans detect signatures of selection along a salinity gradient in populations of the intertidal seaweed *Fucus serratus* on a 12 km scale. *Marine Genomics*, **4**, 41–49.

Hardy FG & Guiry MDR (2003). A Check-list and Atlas of the Seaweeds of Britain and Ireland. British Phycological Society London.

Ingolfsson A (2008). The invasion of the intertidal canopy-forming alga Fucus serratus L. to southwestern Iceland: Possible community effects. *Estuarine Coastal and Shelf Science*, **77**, 484–490.

Malm T & Kautsky L (2003). Differences in life-history characteristics are consistent with the vertical distribution pattern of *Fucus serratus* and *Fucus vesiculosus* (Fucales, Phaeophyceae) in the central Baltic Sea. *Journal of Phycology*, **39**, 880–887.

Malm T, Kautsky L, & Engkvist R (2001). Reproduction, recruitment and geographical distribution of *Fucus serratus* L. in the Baltic Sea. English. *Botanica Marina*, **44**, 101–108.

Metelsty AA & Frolov AA (2008). A comprehensive coastal expedition among bays and gulfs of Kolsky peninsula. *News of IPY. International polar year 2007/08 in the Russian Federation and around the world*, 19–21.

Pearson GA, Lago-Leston A, & Mota C (2009). Frayed at the edges: selective pressure and adaptive response to abiotic stressors are mismatched in low diversity edge populations. *Journal of Ecology*, **97**, 450–462.

3. ***Ascophyllum nodosum***

Åberg P (1992). A demographic study of two populations of the seaweed *Ascophyllum nodosum*. *Ecology*, **73**, 1473–1487.

Ang PO, Sharp GJ, & Semple RE (1993). Changes in the population structure of *Ascophyllum nodosum* (L.) Le Jolis due to mechanical harvesting. *Hydrobiologia*, **260–261**, 321–326.

Araújo R, Serrão EA, Sousa-Pinto I, & Åberg P (2011). Phenotypic differentiation at southern limit borders: The case study of two fucoid macroalgal species with different life-history traits. *Journal of Phycology*, **47**, 451–462.

Araújo R, Vaselli S, Almeida M, Serrao E, & Sousa-Pinto I (2009). Effects of disturbance on marginal populations: human trampling on *Ascophyllum nodosum* assemblages at its southern distribution limit. *Marine Ecology Progress Series*, **378**, 81–92.

Cho GY, Lee SH, & Boo SM (2004). A new brown algal order, Ishigeales (Phaeophyceae), established on the basis of plastid protein coding rbcL, psaA, and psbA region comparisons. *Journal of Phycology*, **40**, 921–936.

Chopin T, Marquis PA, & Belyea EP (1996). Seasonal dynamics of phosphorus and nitrogen contents in the brown alga *Ascophyllum nodusum* (L) Le Jolis, and its associated species *Polysiphonia lanosa* (L.) Tandy and *Pilayella littoralis* (L.) Kjellman, from the Bay of Fundy, Canada. *Botanica Marina*, **39**, 543–552.

Gollety C, Thiebaut E, & Davoult D (2011). Characteristics of the *Ascophyllum nodosum* stands and their associated diversity along the coast of Brittany, France. *Journal of the Marine Biological Association of the United Kingdom*, **91**, 569–577.

Guillaumont B, Callens L, & Dion P (1993). Spatial distribution and quantification of *Fucus* species and *Ascophyllum nodosum* beds in intertidal zones using spot imagery. *Hydrobiologia*, **261**, 297–305.

Datasets accessed through GBIF

1. Botanical Museum, Copenhagen, the Phycology Herbarium
2. Botanical Garden and Museum, the Algae Collection from Denmark, Faroe Islands and Iceland
3. The Seaweeds of Ireland dataset, held by the National Biodiversity Data Centre, www. biodiversityireland.ie
4. British Phycological Society, Seaweed data for Great Britain and Ireland
5. The Rocky Shore Macroalgae dataset, held by the National Biodiversity Data Centre, www.biodiversityireland.ie
6. Joint Nature Conservation Committee, Marine Nature Conservation Review MNCR and associated benthic marine data held and managed by JNCC
7. Scottish Natural Heritage - Marine Nature Conservation Review MNCR and associated benthic marine data held and managed by Scottish Natural Heritage
8. Marine Conservation Society, Seasearch Marine Surveys,
9. Countryside Council for Wales - Littoral Marine data from Countryside Council for Wales (CCW) Technical Support (Research & Monitoring) Contracts, Wales
10. Natural England, Marine Nature Conservation Review (MNCR) and associated benthic marine data held and managed by English Nature
11. North & East Yorkshire Ecological Data Centre, Non-sensitive Records from all taxonomic groups
12. Schatzinsel Norderney, GEO-Tag der Artenvielfalt
13. Artenvielfalt der Nordsee - Helgoland, GEO-Tag der Artenvielfalt
14. Wangerooge, GEO-Tag der Artenvielfalt
15. Artenvielfalt der Nordsee - Sylt, GEO-Tag der Artenvielfalt
16. Wattenmeer-Safari (Wurster Watt), GEO-Tag der Artenvielfalt
17. Gesamtartenliste Bremerhaven Helgoland und Sylt, GEO-Tag der Artenvielfalt

18. Artenvielfalt der Nordsee - Bremerhaven Dorum-Neufeld
19. Tauchen und Meer, GEO-Tag der Artenvielfalt
20. Real Jardin Botanico Madrid: MA-Algae, http://www.rjb.csic.es/herbario/crypto/crypcond.htm
21. Algae S
22. Universidad de Málaga: MGC-Algae, http://webdeptos.uma.es/BiolVeg/04Her/00Hherb/01herb.html
23. Universidad de Oviedo. Departamento de Biología de Organismos y Sistemas: FCO-Algae, http://www.uniovi.es/BOS/Herbario/FCO.htm
24. Herbario del Departamento de Biología Vegetal (Botánica) de la Universidad de La Laguna; Tenerife (TFC)
25. CONN
26. Cumbria Biodiversity Data Centre - Norman and Florence Hammond records, Seawatch and coastal survey records
27. The Norwegian Species Observation Service held by the Norwegian Biodiversity Information Centre, www.artsdatabanken.no
28. AWI-Herbarium Marine Macroalgae
29. Algae Specimens, Agder naturmuseum (KMN)
30. Artdata
31. Algae Norwegian College of Fishery Science

Datasets accessed through OBIS

1. Rapid Assessment Surveys of Native and Introduced Marine Organisms in the Northeast United States, Staten Island New York to Eastport Maine
2. Eelgrass Community across an eutrophication gradient in New Brunswick and Prince Edward Island, Canada
3. BioMar - Ireland: benthic marine species survey
4. Intertidal rocky shore assemblages in Portugal
5. ICES contaminants and biological effects
6. Checklist of benthic marine algae and cyanobacteria of northern Portugal
7. Marine Life List of Ireland
8. Macroalgal communities of intertidal rock pools in Portugal
9. Coastal Habitat Invasives Monitoring Program
