# Supplementary material for: Climate change impact on seaweed meadow distribution in the North Atlantic rocky intertidal
Source: Ecol Evol. 2013 Apr 12;3(5):1356–73. doi: 10.1002/ece3.541 (PMC3678489; doi:10.1002/ece3.541)
Supplement: Supplementary file 4 [file ece30003-1356-SD4.docx]

| **Model** | |  | |  | | **1** | **2** | **3** | **4** | **5** | **6** | **7** | **8** |  |
| --- | --- | --- | --- | --- | --- | --- | --- | --- | --- | --- | --- | --- | --- | --- |
|  | **Variable** | | **Derivative** | | **Unit** |  |  |  |  |  |  |  |  | |
| **Included environmental raster** | DA | | Maximum | | m^-1^ | x | x | x | x | x | x | x |  | |
|  | DA | | Mean | | m^-1^ | x | x |  |  |  |  |  |  | |
|  | DA | | Minimum | | m^-1^ | x |  |  |  |  |  |  |  | |
|  | Dissox | | Mean | | ml/l | x | x | x | x | x |  |  |  | |
|  | Nitrate | | Mean | | µmol/l | x | x | x |  |  |  |  |  | |
|  | PAR | | Maximum | | Einstein  /m^2^/day | x |  |  |  |  |  |  |  | |
|  | PAR | | Mean | | Einstein  /m^2^/day | x |  |  |  |  |  |  |  | |
|  | pH | | Mean | | - | x |  |  |  |  |  |  |  | |
|  | Phos-phate | | Mean | | µmol/l | x |  |  |  |  |  |  |  | |
|  | **Salinity** | | **Mean** | | **PSS** | **x** | **x** | **x** | **x** | **x** | **x** | **x** | **x** | |
|  | SAT | | Maximum | | °C | x | x | x | x | x | x |  |  | |
|  | SAT | | Mean | | °C | x |  |  |  |  |  |  |  | |
|  | **SAT** | | **Minimum** | | **°C** | **x** | **x** | **x** | **x** | **x** | **x** | **x** | **x** | |
|  | SAT | | Range | | °C | x | x | x |  |  |  |  |  | |
|  | Silicate | | Mean | | µmol/l | x |  |  |  |  |  |  |  | |
|  | SST | | Maximum | | °C | x |  |  |  |  |  |  |  | |
|  | SST | | Mean | | °C | x | x |  |  |  |  |  |  | |
|  | **SST** | | **Minimum** | | **°C** | **x** | **x** | **x** | **x** | **x** | **x** | **x** | **x** | |
|  | SST | | Range | | °C | x | x | x | x |  |  |  |  | |
| **Perfor-mance** | BIC |  | |  | | 5994 | 6187 | 6238 | 6146 | 6067 | 6053 | 5939 | 5828 | |
|  | AICc |  | |  | | 6277 | 6104 | 6099 | 5990 | 5896 | 5884 | 5777 | 5671 | |
