# Supplementary figures and images for: Climate change impact on seaweed meadow distribution in the North Atlantic rocky intertidal
Source: Ecol Evol. 2013 Apr 12;3(5):1356–73. doi: 10.1002/ece3.541 (PMC3678489; doi:10.1002/ece3.541)

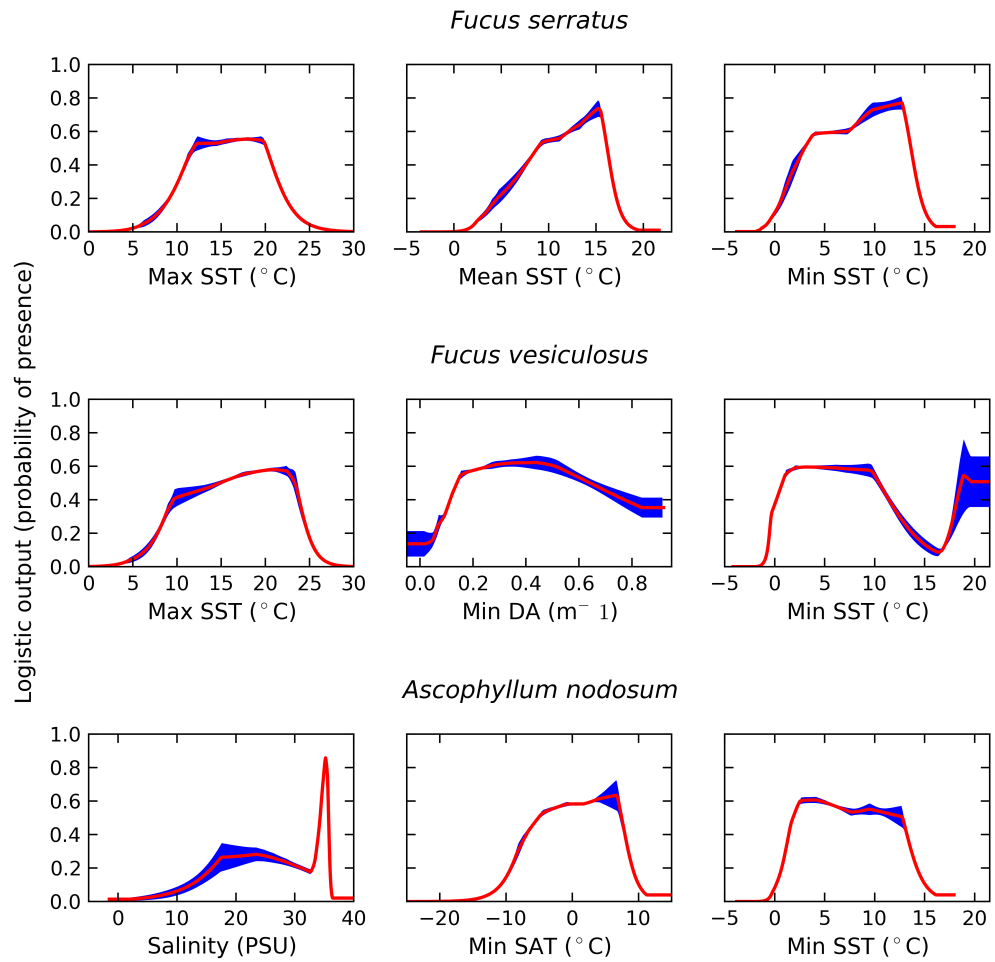

Supplement: Supplementary file 5 [file ece30003-1356-SD5.pdf]

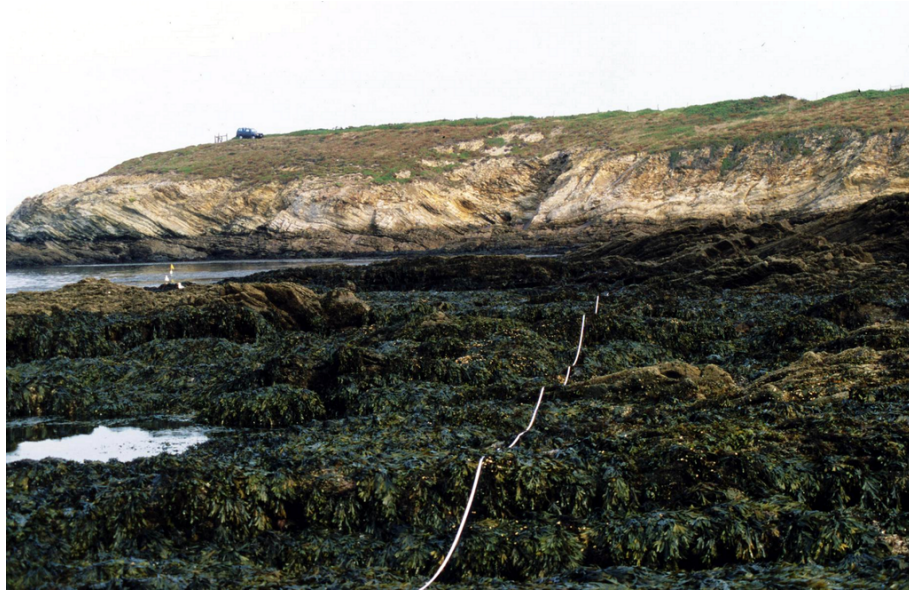

(a) 1999

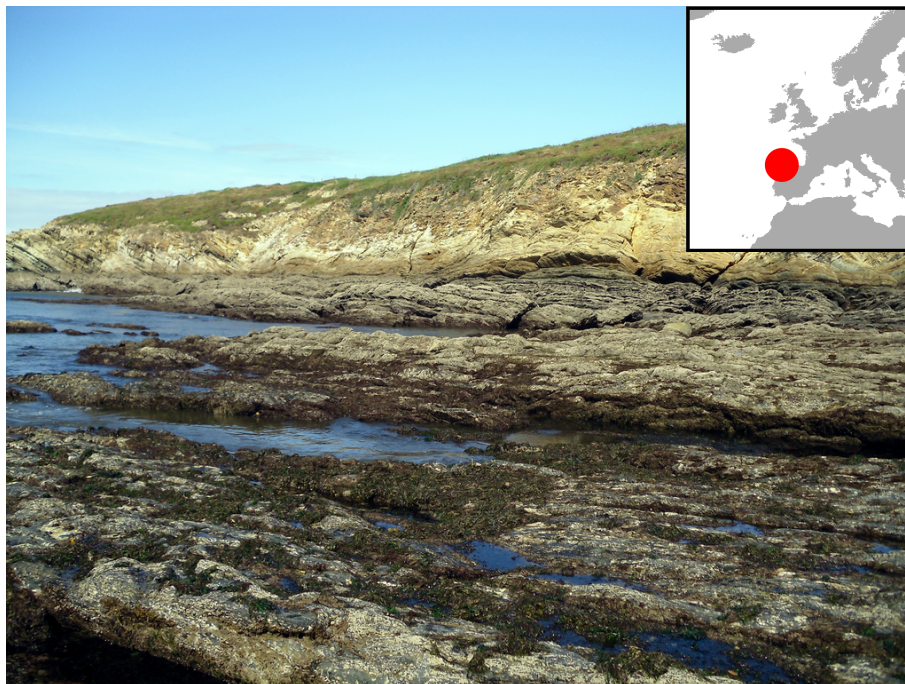

(b) 2010

Supplement: Supplementary file 6 [file ece30003-1356-SD6.pdf]
